# Impact of Alcohol Abuse on Susceptibility to Rare Neurodegenerative Diseases

**DOI:** 10.3389/fmolb.2021.643273

**Published:** 2021-06-09

**Authors:** Iskra Araujo, Amy Henriksen, Joshua Gamsby, Danielle Gulick

**Affiliations:** ^1^ Gulick Laboratory, Byrd Neuroscience Institute, University of South Florida Health, Tampa, FL, United States; ^2^ Department of Molecular Medicine, Morsani College of Medicine, University of South FL, Tampa, FL, United States

**Keywords:** alcoholism, addiction, aging, ataxia, dementia, stress, Epigenetics, inflammation

## Abstract

Despite the prevalence and well-recognized adverse effects of prenatal alcohol exposure and alcohol use disorder in the causation of numerous diseases, their potential roles in the etiology of neurodegenerative diseases remain poorly characterized. This is especially true of the rare neurodegenerative diseases, for which small population sizes make it difficult to conduct broad studies of specific etiological factors. Nonetheless, alcohol has potent and long-lasting effects on neurodegenerative substrates, at both the cellular and systems levels. This review highlights the general effects of alcohol in the brain that contribute to neurodegeneration across diseases, and then focuses on specific diseases in which alcohol exposure is likely to play a major role. These specific diseases include dementias (alcohol-induced, frontotemporal, and Korsakoff syndrome), ataxias (cerebellar and frontal), and Niemann-Pick disease (primarily a Type B variant and Type C). We conclude that there is ample evidence to support a role of alcohol abuse in the etiology of these diseases, but more work is needed to identify the primary mechanisms of alcohol’s effects.

## Introduction

### Alcohol Addiction and Aging

Alcohol use disorders (AUDs) and risky drinking (including adolescent and *in utero* exposure to alcohol) present a major social, financial, and medical burden in the United States despite the campaigns and preventative efforts meant to reduce them. Alcohol is the most commonly abused drug across the lifespan by far (Schulte and Hser, 2014). According to 2015 data from The National Survey on Drug Use and Health in the United States, 138 million individuals consume alcohol on a regular basis in the United States, with almost half of drinkers reporting problem (binge or heavy) alcohol consumption, and 15.7 million reporting an AUD. By contrast, only 64 million respondents reported past month tobacco use, 22 million reported past month marijuana use, and less than 13 million reported past month use of other illicit drugs. An additional 2.7 million people reported an SUD co-occurring with an AUD (Administration, 2015). The sheer prevalence of problem drinking makes alcohol exposure a common etiological factor in a wide range of diseases. In this review, we will discuss the evidence for a role of alcohol exposure in the etiology of rare neurodegenerative diseases, as well as mitigating factors, such as, age of exposure, gender, and alcohol consumption pattern.

Most research into AUDs has focused on fetal alcohol, adolescent binge drinking, and chronic drinking in early adulthood. However, increasing life expectancy and greater independence throughout the geriatric period has made alcohol addiction in elderly adults a growing issue (Simoni-Wastila and Yang, 2006). Approximately 40% of adults over 65 drink alcohol, and almost 5% of these older adults struggle with an AUD (Administration, 2015). Furthermore, alcohol abuse is reported in 50% of geriatric psychiatry ward admissions (compared to 30% of general geriatric medical admissions (Caputo et al., 2012)). Thus, we will discuss the shared and unique effects of alcohol at each stage of human—and neural—development.

Recent research has suggested that as many as 1 in every 10 children suffers from a fetal alcohol spectrum disorder (FASD), and at least 10% of these children suffer from the most severe form of FASD, fetal alcohol syndrome (FAS) (May et al., 2018). All children with FASD present with some degree of growth restriction and neurodevelopmental abnormalities (Stratton et al., 1996). Unlike children with FAS, however, children with milder FASD frequently lack the facial dysmorphology that can drive a clinical diagnosis, and thus FASD goes drastically under-reported (Sokol et al., 2003). Perhaps surprisingly, even at the fetal stage, there is a significant sex difference in susceptibility to FAS, with male children experiencing more devastating effects (Terasaki et al., 2016). During gestation, the brain is undergoing rapid proliferation and synaptogenesis, and neural connectivity is therefore highly sensitive to any environmental disruptions. Fetal alcohol exposure activates microglia during gestation, impacting the developing brain, but also sensitizing the immune system, leading to a hyperactive inflammatory response and associated cognitive deficits in response to stress in adulthood (Terasaki and Schwarz, 2016). Fetal alcohol exposure also impairs autophagy (Girault et al., 2017), and activation of TLR4 is necessary to generate the neurodevelopment changes and behavioral symptoms of fetal alcohol exposure (Shukla et al., 2018). These changes impact neuroinflammation (Pascual et al., 2017; Noor and Milligan, 2018), oxidative stress (Bhatia et al., 2019; Brocardo et al., 2011), and cognition. At the same time, fetal alcohol exposure has been shown to permanently disrupt the endogenous hypothalamus-pituitary-adrenal (HPA) rhythm mediating cyclic cortisol release, suggesting a long-term alteration in stress reactivity and signaling (Ouellet-Morin et al., 2011). Considering the permanent, widespread neuropathological effects of fetal alcohol exposure (Lo et al., 2017; Kleiber et al., 2014; Guizzetti et al., 2014), even this early experience is likely to increase susceptibility to neurodegenerative disease.

Adolescence is the second critical period during which alcohol produces severe neurodevelopmental changes. By 12th grade, at least 75% of teenagers in the United States have tried alcohol (Windle, 2003; (Wheaton et al., 2016), and more than 5 million 11- to 17-year-olds engage in binge drinking each year. Exaggerating this effect, and complicating the interpretation of adolescent alcohol effects, 45% of adults who began drinking in adolescence go on to meet the criteria for alcohol dependence later in life (NIAAA, 2010; (Hasin et al., 2007). Teenagers tend to find alcohol more stimulatory (Treloar et al., 2017), and they are less sensitive to the negative effects of alcohol (Spear, 2011), leading them to drink larger volumes of alcohol in a single sitting. Unlike in fetal alcohol exposure, in adolescence, there is no statistical difference between sexes in total alcohol intake or binge drinking (Administration, 2019), suggesting that both sexes are at similar risk for long-term damage due to alcohol misuse. However, although alcohol slows the transition from immature to mature synaptic plasticity and neural networking in both sexes (Crews et al., 2016), there are a number of sex differences in the alcohol-induced changes in neurotransmission and stress responding (Flores-Bonilla and Richardson, 2020).

Unlike adolescent alcohol consumption, there are major differences in the drinking habits of adult men and women. Men are more likely to struggle with an AUD than women, perhaps because women experience more negative outcomes as a result of AUD (McHugh et al., 2018). Furthermore, there are sex differences in susceptibility to alcohol-induced pathology in adulthood as well (Savage et al., 2000), including differences in neurodegeneration in the HPA axis (Silva et al., 2009) and hippocampus (Devaud et al., 2020; (Maynard et al., 2018). Beyond the effects of sex, another complication in interpreting the effects of adult consumption on later neurodegeneration is the pattern of alcohol intake. There are some data to support a neuroprotective effect of moderate alcohol consumption on neurodegeneration and cognitive function (Collins et al., 2009; Pignataro, 2019). On the other hand, heavier alcohol consumption—regardless of whether it meets the criteria for binge drinking—is universally associated with both neurobiological and behavioral deficits. Whereas most problem drinking in adolescence follows a binge pattern, adult problem drinking consists of both binge drinking (∼26% of the United States population) and the more severe chronic, heavy consumption (∼7%) (Administration, 2019). Chronic alcohol is associated with global neurodegeneration, largely through oxidative stress and inflammation, that causes greatest damage in the prefrontal cortex (Crews and Vetreno, 2014), and binge alcohol drinking is associated with a decrease in hippocampal proliferation and survival of neural stem cells (Nixon and Crews, 2002). Thus, alcohol can affect neuron density at both the conception and destruction of neurons—by reducing neurogenesis and by driving neuron death. Where feasible, we will discuss the effects of specific drinking patterns independently; however, most self-reported histories of alcohol intake fail to differentiate different types of problem drinking.

There are few studies that evaluate the effects of problem drinking in geriatric patients. We know that AUDs are on the rise in the geriatric population due to a rapidly aging population and the fact that the elderly do not need to drink as much to experience the negative consequences of alcohol (Caputo et al., 2012; Oslin, 2004). Furthermore, the sex differences in alcohol intake that are observed in earlier adulthood disappear in the aging population, at least in part due to isolation and depression-induced alcohol seeking in older women (Helvik et al., 2020; Roche et al., 2020). From animal models, we know that the hippocampal immune response to alcohol is exacerbated in the aging brain, largely due to changes in the neuroinflammatory response (Kane et al., 2014; Gano et al., 2017).

Not only does alcohol misuse damage physical and mental health, lifestyle, and family life (Donovan et al., 2005), recent studies suggest that a history of AUD is a major risk factor for common neurodegenerative disorders, such as, AD and related dementias, but not for some other neurodegenerative diseases (Schwarzinger et al., 2018; Venkataraman et al., 2016; Heymann et al., 2016; Tyas, 1996; Xue et al., 2017). Specifically, regular alcohol consumption averaging around 3 drinks per day increases the risk of neurodegenerative dementias (Xu et al., 2017), whereas there is no association between alcohol intake and the risk of developing Parkinson’s disease (Jiménez-Jiménez et al.,; Kim et al., 2020); and only a conditional association in amyotrophic lateral sclerosis (D'Ovidio et al., 2019). As many as 65% of adults report a history of either AUD or PAE, yet only about 10% of the individuals struggling with AUD will seek treatment, and there are many obstacles to long-term abstinence e.g. poor compliance, withdrawal symptoms, treatment side effects; (Azevedo and Mammis, 2018). Even contingency management, the most effective intervention for AUD (Gao et al., 2018), has less than a 50% success rate (Dougherty et al., 2015). Thus, millions of individuals are putting themselves at risk of alcohol-induced brain damage. Alcohol is the primary driver of some neurodegenerative diseases, such as, Wernicke–Korsakoff syndrome and certain forms of ataxia (de la Monte and Kril, 2014). In addition, AUD is a significant predictor of AD (odds ratio 3–4) (Zilkens et al., 2014; Stephen et al., 2019); that also accelerates the onset of AD symptoms (Harwood et al., 2010). Considering that AD shares a number of pathological features with rare neurodegenerative diseases, it is likely that alcohol plays a broader role in the etiology of these diseases as well.

### Alcohol Use and Rare Neurodegenerative Diseases

**FIGURE 1:**
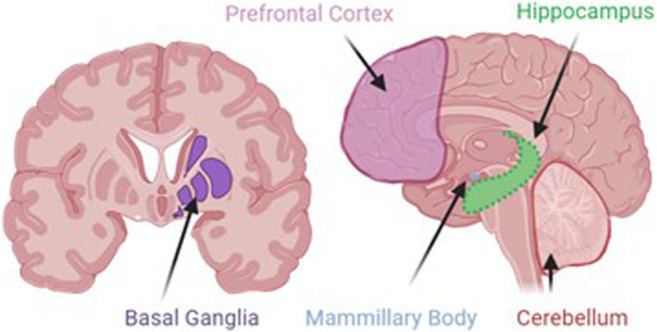
Although alcohol has effects on a number of ubiquitous cellular processes, such as, autophagy, inflammation, and stress, the localization of alcohol-induced neurodegeneration is likely to be specific to areas where alcohol produces its greatest effects. These areas include the prefrontal cortex, the site of working memory and executive function processes; basal ganglia, the dopaminergic site of motor initiation and habitual behavior; the mammillary bodies, the hypothalamic nuclei that integrate with the emotion and memory centers of the limbic system; the hippocampus, the site of declarative memory consolidation; and the cerebellum, the site of motor coordination and balance.

**FIGURE 2:**
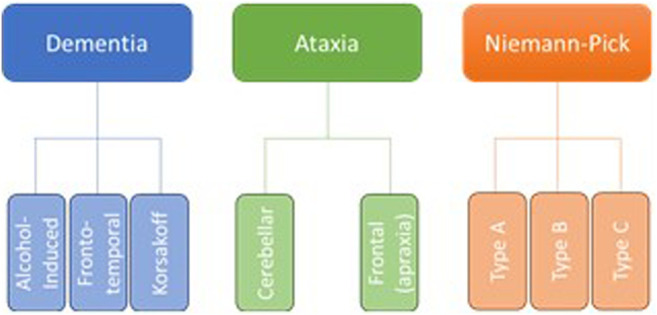
Although there are dozens of rare neurodegenerative disorders, we have chosen to focus on those in which the etiology suggests an increased susceptibility to the effects of alcohol abuse, as well as those in which at least some research has been done to suggest a direct effect of alcohol on neurodegeneration.

Neurodegeneration is the progressive loss of neuronal structure or function and can occur at any age, and in any region of the nervous system. For example, neurodegeneration is most prominent in the hippocampus of patients with Alzheimer’s disease, in the basal ganglia of patients with Parkinson’s disease, and in the upper and lower motor neurons of patients with amyotrophic lateral sclerosis. Neurodegeneration is similarly variable in the rare neurodegenerative diseases, and in this review, we will highlight other brain regions that are most likely to be compromised by a history of problem drinking, including the prefrontal cortex, hypothalamus, hippocampus, and cerebellum (Figure 1). We will focus on mechanisms of action through which alcohol is likely to drive neurodegeneration in a variety of diseases; these mechanisms include neuroinflammation and oxidative stress, autophagy, and transcriptomic or methylomic changes in gene expression (Table 1). We will also discuss the potential impact of alcohol’s effects on the stress response and on canonical neurodegenerative proteinopathies, such as, tau pathogenesis. In order to remain within the bounds of feasibility, we have examined the putative role of alcohol in rare neurodegenerative diseases from three broad categories: dementias (alcohol-related or -induced, frontotemporal, and Wernicke-Korsakoff syndrome), ataxias (cerebellar, frontal, sensory), and Niemann-Pick Types B and C (Figure 2).

**TABLE 1:** Major targets of alcohol that contribute to the pathophysiology of neurodegeneration.

Pathway	Alcohol target	Pathological role	References
Alcohol metabolism	Alcohol/aldehyde dehydrogenases	Oxidative damage	Crabb et al. (2004)
Apoptosis	Voltage-dependent anion channel 1	Mitochondrial dysfunction	Abulaiti et al. (2016)
Autophagy	mTOR1	Failure to clear toxic proteins	Matsushita et al. (2008)
Epigenetic	DNA methylases	Various	Nieratschker et al. (2013)
Epigenetic	Histone deacetylase	Various	Pandey et al. (2008)
Immune	Toll-like receptor 4	Inflammation	Alfonso-Loeches et al. (2010)
Immune	High mobility group protein 1	Inflammation	Vetreno and Crews (2012)
Neurotransmission	Glutamate NMDA receptors	Excitotoxicity	Wiegmann et al. (2020)
Neurotransmission	GABAA receptors	Excitotoxicity	Wiegmann et al. (2020)
Neurotrophins	BDNF and receptors	Cell survival	Sakai et al. (2005)
White matter	Calpain	Myelin degradation	Samantaray et al. (2015)
White matter	Acetaldehyde protein adducts	Axonal damage	Niemelä (2001)

Table-specific references Sakai et al. (2005) and Crabb et al. (2004).

Unsurprisingly, there are numerous studies evaluating alcohol-related dementia. As defined by the Centers for Disease Control, dementia is an impairment in the ability to remember, think, or make decisions that goes beyond the decline seen in normal aging (Promotion, National Center for Chronic Disease Prevention and Health, 2019). Alcohol-related dementia indicates any case in which the dementia is exacerbated by alcohol abuse, but not caused by it. In contrast, cases in which alcohol is a causative factor, such as, in some cases of vascular and frontotemporal dementia, are not included in this definition. Many early onset cases of dementia can be classified as either alcohol-related or as a sequela of a history of AUD (Rehm et al., 2019; Schwarzinger et al., 2018).

**FIGURE 3:**
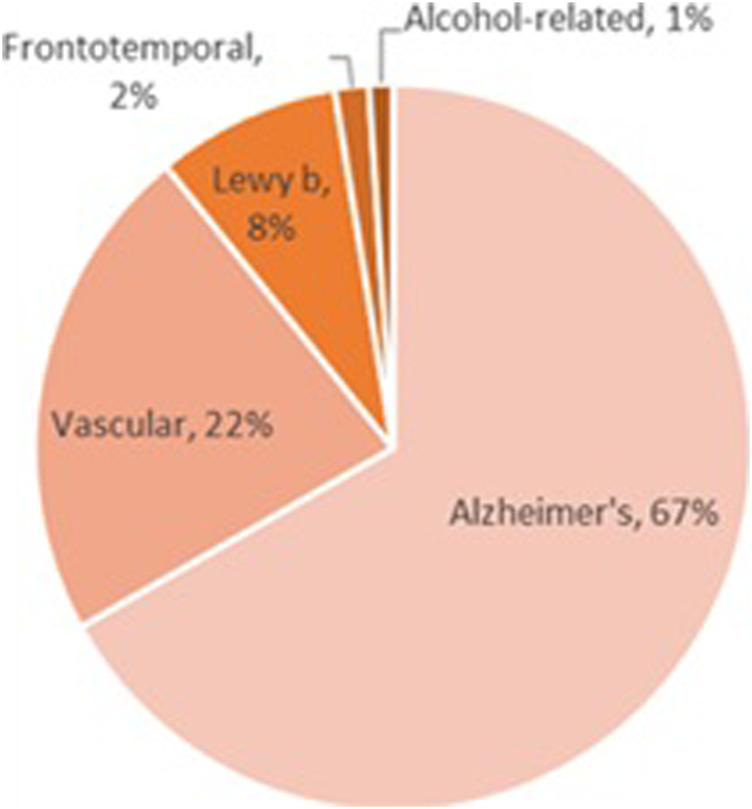
Most of the work examining the role of alcohol exposure on dementia has been in the field of Korsakoff syndrome; despite the rarity of this disease, alcohol is almost exclusively the culprit in the cases that have been identified. Alcohol-induced dementia is even less common, although alcohol-related dementias (those in which alcohol is one of multiple etiological factors) occur across all dementia subtypes. In addition, frontotemporal dementia occurs due to neurodegeneration in areas that are most heavily impacted by alcohol exposure; although little work has been done to examine the role of alcohol in the etiology of frontotemporal dementia, this topic deserves further study.

Despite the relatively small number of alcohol-related dementia cases (Figure 3), alcohol is likely a factor in hundreds of thousands of other dementia cases. For example, alcohol-induced changes in cerebral blood flow and vessel integrity may drive vascular dementia, and alcohol’s effects on amyloidosis and clearance mechanisms may drive Alzheimer’s disease (Xu et al., 2017). However, alcohol can have a more direct effect in frontotemporal dementia, as alcohol-induced neurodegeneration is often localized to the prefrontal cortex.

The final form of alcohol-related dementia is Wernicke–Korsakoff syndrome. This syndrome is comprised of a trio of independent symptoms (confusion and cognitive impairment, extraocular motor deficits, and ataxia) that are caused by a deficiency in thiamine, also known as vitamin B1, and this deficiency is almost always secondary to chronic alcoholism. The development of the syndrome can be divided into the initial stage of Wernicke encephalopathy, which can be reversed with thiamine replacement therapy, and the permanent neurodegeneration and behavioral deficits that produce Korsakoff syndrome when patients fail to receive thiamine replacement (Arts et al., 2017). The latter will be examined in this review, as it is only with these irreversible changes that neurodegeneration is observed.

Ataxia is defined as the loss of control over body movements due to difficulties with balance or coordination. Although ataxia is an endophenotype noted in some forms of dementia, including Wernicke-Korsakoff syndrome, ataxias also make up a distinct category of neurodegenerative motor disorders. Many ataxias can be traced to specific genetic factors; this review will focus only on those in which alcohol is likely to present as an etiological factor. The most common form of ataxia by far is cerebellar, but alcohol’s effects may extend to include ataxias that can be traced to damage in other brain regions where alcohol produces severe damage, including the basal ganglia (frontal ataxia), and in peripheral nerves (sensory ataxia) (Fein and Greenstein, 2013). Although this review focuses on alcohol’s effects in the central nervous system, we will briefly touch on sensory ataxia in comparison to the central motor effects of alcohol.

Finally, this review will discuss the potential role of alcohol in specific subtypes of Niemann-Pick disease. Niemann-Pick describes three related, inherited disorders of lipid metabolism. Although an inherited disorder, the mutation associated with Niemann-Pick Types A and B is associated with high levels of alcohol intake in an animal model (Kalinichenko et al., 2019b), and the impact of alcohol on metabolism is likely to exacerbate the damage due to oxidative stress in Type C (Kalinichenko et al., 2019b). Far less work has been done in this area than in the first two areas to be discussed, but the work that has been done suggests that alcohol should be considered as a contributing factor here as well.

## General Effects of Alcohol in Neurodegeneration

Although most of our understanding of the mechanisms by which alcohol impacts neurodegeneration have been gleaned from studies in the most common neurodegenerative diseases, some preliminary work has been done in rare neurodegenerative disease models and clinical cases as well. Thus, we will briefly review the mechanisms of alcohol-induced neuronal damage that have been revealed in common neurodegenerative diseases, and the applicability of these mechanisms to the rare neurodegenerative diseases. Finally, we will explore the work that has been done to better understand the role of alcohol, specifically in each rare neurodegenerative disease.

**FIGURE 4:**
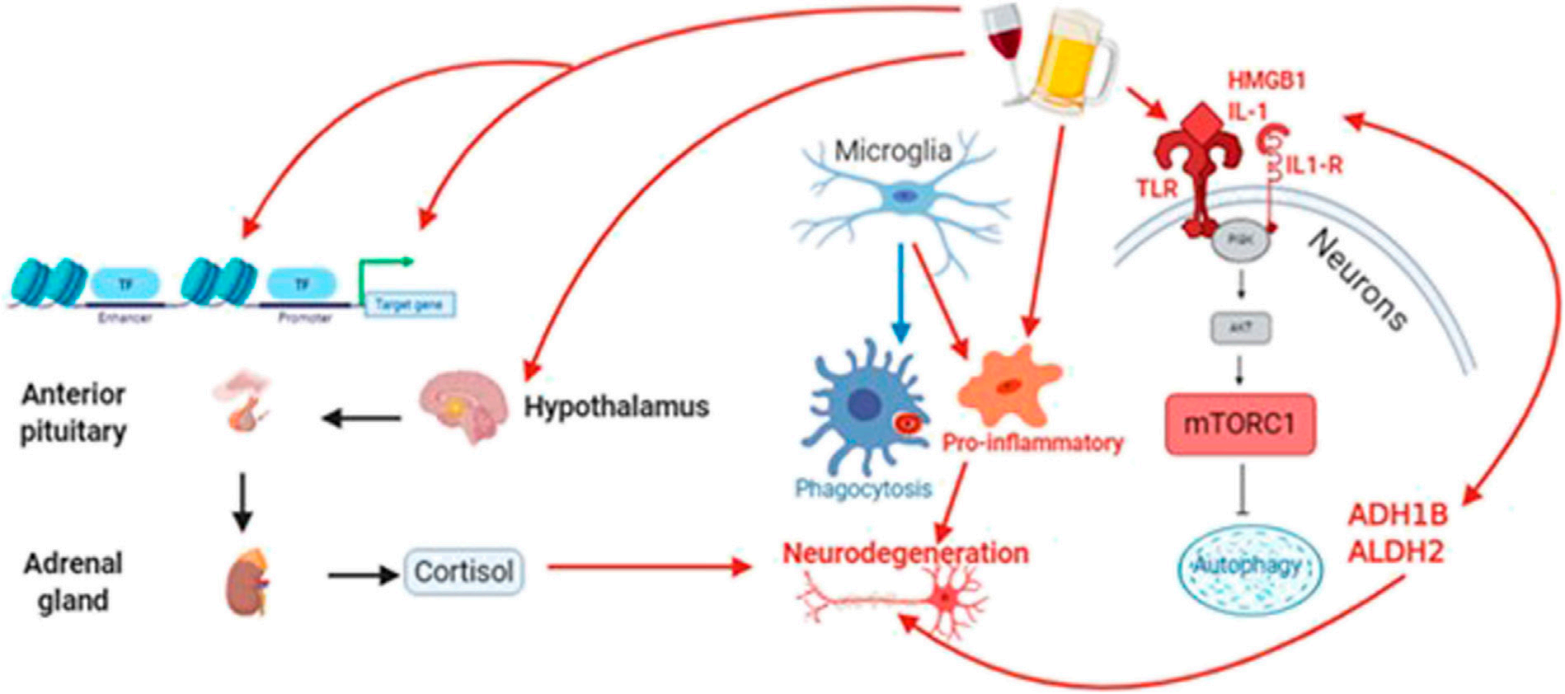
Alcohol acts through diverse signaling pathways, including oxidative stress, to increase neuroinflammation and reduce both autophagic and cytophagic clearance of toxic protein species that contribute to neurodegeneration. At the same time, alcohol alters the expression and activity of epigenetic proteins (e.g., methyltransferases/demethylases and DNA acetylases/deacetylases. Finally, alcohol drives an unhealthy stress response through the hypothalamic-pituitary-adrenal (HPA) axis, leading to long-term increases in circulating cortisol.

The largest body of work into the clinical correlates of alcohol-induced neurodegeneration, outside of Wernicke–Korsakoff syndrome, belongs to studies of Parkinson’s disease and Alzheimer’s disease. And although the most heavily impacted brain areas differ between these diseases, as well as across the rare neurodegenerative diseases, the mechanisms of alcohol’s actions are likely to be redundant (Figure 4 is provided as an overview of these actions). For example, chronic alcohol abuse is associated with both axonal demyelination and degeneration in multiple brain regions (Harper and Matsumoto, 2005; Harper et al., 1985); because alcohol disrupts the lipid homeostasis that is essential for myelin synthesis and maintenance (Yalcin et al., 2017) and drives calpain, a neutral protease (Samantaray et al., 2015). Such a white matter defect is likely to cause behavioral symptoms in a spectrum of diseases, although the specific symptoms will depend on the nature of each disease—alcohol will have a greater effect in regions already damaged by disease processes.

### Oxidative Stress

Neurodegenerative diseases are comprised of variable loss of neuronal and synaptic features, yet there are also common pathogenic features, such as, oxidative stress, inflammation, and protein aggregation. Common pathologies associated with neurodegenerative diseases, such as, Alzheimer’s disease and many of the rarer neurodegenerative diseases often feed into one another, creating a self-perpetuating cycle of neuronal damage. These pathologies, such as, protein hyperphosphorylation, deficits in protein clearance, and pro-inflammatory signals are all associated with oxidative stress. Oxidative stress, such as, that produced when the metabolism of alcohol to acetaldehyde and then to acetate generates reactive oxygen species (ROS) and nitric oxide (NO), damages mitochondria and DNA itself. An inflammatory response is triggered by the mitochondria due to the disruption in oxidative phosphorylation (Cenini et al., 2019; Picca et al., 2020). This inflammatory response result in the activation of pro-inflammatory cytokines, microglia, and astrocytes, resulting in the suppression of neurogenesis and the hyperphosphorylation of both tau and Aβ, two canonical components of neurodegeneration (López-Armada et al., 2013). Commonly, this cellular damage also initiates apoptotic and necrotic processes in neurons (Singh et al., 2019) and loss of CREB transcription, which is essential to programmed neuron survival (Crews and Nixon, 2009). At the same time, acetaldehyde produces adducts that can cause white matter and axonal damage (Niemelä, 2001) Since oxidative stress is produced by the breakdown of alcohol into acetaldehyde and the further breakdown of acetaldehyde into acetate, the greater the amount of alcohol consumed, especially in a binge drinking episode, the greater the oxidative stress in the brain.

### Proteinopathy

Although best characterized in Alzheimer’s disease, both amyloidosis and tauopathy are common to the pathological cascades across a range of neurodegenerative disorders. Tau protein is a microtubule polymerization promoter protein which is induced in nerve cell differentiation, allowing for assembly and stabilization of microtubules that contribute to the proper structure and function of neurons. This protein comes from the microtubule-associated protein (MAP) family and is mainly expressed by neuronal cells, especially localized to the axon (Guzman-Martinez et al., 2019). Its structure promotes the action of secondary kinases, resulting in production of new epitopes. In the case of pathological tau hyperphosphorylation, alterations in structure or function of tau in neuron leads to the production of neuronal fibrillary tangles. These tangles are associated with axonal degeneration in neurons. Amyloidosis is characterized by the extracellular accumulation of Aβ plaques, which can then drive the intracellular accumulation of neurofibrillary tangles. Accumulation of Aβ42/43 results in self-aggregation and extracellular plaques. In rats, this accumulation has been shown to result in tau hyperphosphorylation and subsequent neuritic dystrophy (Selkoe and Hardy, 2016). Positive amyloid PET scan is also associated with high CSF concentrations of tau, decreased glucose metabolism, and brain atrophy (Selkoe and Hardy, 2016). Alcohol exacerbates amyloidosis (Huang et al., 2017; Self et al., 2005); and delays clearance of hyper-phosphorylated tau (pTau), leading to aggregation and cytotoxicity (Gendron et al., 2008). Prolonged AUD causes Wernicke encephalopathy due to thiamine deficiency, increasing CSF levels of both Aβ and total tau beyond levels seen even in AD patients (Matsushita et al., 2008). Although Parkinson’s disease is outside the scope of this review, it is important to note that proteinopathy that underlies this neurodegenerative disease, α-synuclein, is heavily upregulated by chronic alcohol exposure as well (Janeczek and Lewohl, 2017; Janeczek and Lewohl, 2013). Normally, Aβ is processed by two proteases and exported to the cerebrospinal fluid, where microglia phagocytose the toxic protein.

### Protein Clearance

Two essential clearance mechanisms for both toxic proteins include microglial phagocytosis (Gabande-Rodriguez et al., 2020) and neuronal autophagy (Liu and Li, 2019). Perhaps unsurprisingly, alcohol exposure has negative effects on both processes. Alcohol exposure up-regulates the activity of the mTOR1 complex (mTORC1) by phosphorylating mTOR in the brain (Matsushita et al., 2008), which feeds forward to inhibit autophagy (Lee et al., 2017; Menk et al., 2018); and induce apoptosis (Li and Ren, 2007). Not surprisingly, mTORC1 is hyperactive in both aging and neurodegenerative diseases (Perluigi et al., 2015), and recent studies suggest that Aβ increases tau phosphorylation through mTORC1 Incubation with alcohol drives microglia to their pro-inflammatory state and down-regulates phagocytotic activity (Kalinin et al., 2018) by activating toll-like receptors (TLRs) (Fernandez-Lizarbe et al., 2013), especially TLR4 (Alfonso-Loeches et al., 2010). TLRs are recognized as key mediators of AUD-induced neurodegeneration (Yang et al., 2014), and likely provide one mechanism by which both fetal alcohol exposure and binge alcohol drinking contribute to neurodegenerative diseases. A binge episode of alcohol exposure is sufficient to activate microglia and maintain their pro-inflammatory state for 2 weeks after the end of the binge (Shukla et al., 2018; Girault et al., 2017), suggesting that short-term abstinence is not a guarantee of recovery. Prenatal alcohol exposure activates microglia during gestation, impacting the developing brain, but also sensitizing the immune system, leading to a hyperactive inflammatory response and associated cognitive deficits in response to stress in adulthood (Terasaki and Schwarz, 2016). Prenatal alcohol also impairs autophagy (Girault et al., 2017), and activation of TLR4 is necessary to generate the neurodevelopment changes and behavioral symptoms of FAS (Shukla et al., 2018). The combination of alcohol’s effects on microglia and neurons can be extrapolated to severely impair clearance mechanisms for any toxic protein species across all proteinopathies.

### Inflammation and the Immune Response

Binge-like alcohol exposure is sufficient to up-regulate both TLRs and high-mobility group box 1 (HMGB1) (Vetreno and Crews, 2012), or amphoterin, a nuclear protein which can act intracellulary and extracellularly to promote cytokine release and inflammation. At the same time, alcohol-induced oxidative stress and its sequelae, including activation of microglia and astrocytes, cause the release of both Aβ and the same pro-inflammatory cytokines, such as, TNF-a, IL-1B, and IL-6 from neurons (Tzioras et al., 2019). Thus, alcohol acts through multiple pathways to exacerbate the inflammatory response in the brain. This inflammatory response leads to the further production of ROS and NO, resulting in uncontrolled inflammation, and initiating or exacerbating neurodegeneration (Tzioras et al., 2019; (Guzman-Martinez et al., 2019). For a more detailed review of the effects of alcohol on neuroinflammation and immune signaling, please see recent work by Crews and colleagues (Crews et al., 2017) and Erickson and colleagues (Erickson et al., 2019).

### Epigenetics

One of the most prominent physiological mechanisms underlying long-term changes in physiology and susceptibility to disease is the modification of the epigenome. There are a host of aging- and disease-related changes in both DNA and histones. The most well-understood of these modifications are DNA methylation/demethylation and histone acetylation/deacetylation. These changes are essential to signal neuron death following ischemic injury (Noh et al., 2012), as well as in both Alzheimer’s disease and less common dementias, such as, frontotemporal dementia (Lu et al., 2014), as well as in Huntington disease (Buckley et al., 2010). And alcohol produces a slew of changes in the neuronal and glial epigenome (Ponomarev, 2013), and specifically in gene expression patterns. In a transcriptomic comparison between the alcoholic human brain and control brains, the transcript levels of a host of disease-associated genes—including APP, MAPT, BACE, and TREM2—were altered by lifetime alcohol consumption (Farris et al., 2015). In translational work, chronic alcohol is also associated with changes in the transcriptomic profile of the oxidative stress pathway (Bogenpohl et al., 2019). Prenatal alcohol has even more severe effects on the transcription of multiple targets within pathways that mediate neuronal migration and synaptic function (Fischer et al., 2019).

Additionally, the same pathways that show altered transcriptional profiles following alcohol exposure also show a differential epigenetic response to alcohol. By far, the most common epigenetic signature of alcohol is DNA methylation at CpG sites (Nieratschker et al., 2013), although alcohol also reduces histone deacetylase (HDAC) in the limbic regions of the brain (Pandey et al., 2008), which may be relevant to affective symptoms of neurodegenerative diseases. Targets of differential methylation at these sites following alcohol exposure include inflammation (Weng et al., 2015), microglial function, acetaldehyde metabolism, and circadian sleep rhythms (Lussier et al., 2018). Interestingly, chronic alcohol abuse is associated with an accelerated aging epigenotype (Luo et al., 2020). Aging is an essential component to many neurodegenerative diseases; considering that age disrupts essential homeostatic epigenetic processes, such as, the balance between m6A methylase/demethylase activities of METTL3 and FTO (Reitz et al., 2012), alcohol may accelerate neurodegeneration by driving an early aging phenotype.

### The Stress Response

In addition to the myriad effects on intracellular stress in neurodegeneration, there are also effects of the systemic stress response, mediated by the HPA axis and cortisol, that can impact neuronal health and disease. Higher basal levels of cortisol predict a faster decline and greater symptoms of cognitive dysfunction in the mild cognitive impairment stage of Alzheimer’s disease (Popp et al., 2015). Mechanistically, both psychosocial stress and direct application of cortisol increase the hyperphosphorylation of tau, the expression of disease-related tau epitopes, and the formation of neurofibrillary tangles (Sierra-Fonseca and Gosselink, 2018). Similarly, both psychosocial stress and direct application of cortisol increase the amyloid burden in a wide spectrum of animal models (Justice, 2018). Chronic stress also reduces autophagy (Puri and Subramanyam, 2019) and increases pro-inflammatory signaling (Liu Y. Z. et al., 2017), affecting oxidative stress and mitochondrial function (Jeanneteau et al., 2018; (Liu Y. Z. et al., 2017). Long-term effects of early and midlife-stress may persist throughout the lifetime through epigenetic modifications, such as, changes in DNA methylation in histone modification (Rodrigues et al., 2015; Stankiewicz et al., 2013). In addition, microglial function is heavily impacted by psychosocial stress, and long-term shifts in microglial physiology may also explain the lifelong effects on early stress on susceptibility to neurodegenerative disorders (Madore et al., 2020). Considering that alcohol abuse is commonly associated with mood disorders, such as, Major Depressive Disorder, and that alcohol abuse itself can lead to interpersonal, professional, and financial struggles that contribute to psychosocial stress, we hypothesize that the impact of alcohol on neurodegeneration is exacerbated by the activation of the HPA stress axis and the chronic stress response.

## Putative Roles of Alcohol in Specific Rare Neurodegenerative Diseases

### Dementias

As described previously in this review, excessive alcohol use often produces both structural and functional deficits in neural circuits which can contribute to a host of rare neurodegenerative diseases. However, a history of alcohol abuse can also lead to alcohol-induced dementia, in which alcohol exposure is the primary etiological factor. This disease is characterized by numerous cognitive deficits (Angela Maria Ribeiro and Castanheira Pereira, 2011). The most prominent impairments are in learning and memory and in visuospatial function, and these deficits are at least partially recovered if abstinence is maintained (Sachdeva et al., 2016), suggesting transient effects of alcohol rather than neurodegeneration. However, persistent alcohol abuse results in irreversible neurodegenerative damage. Since alcohol-induced vs. -related disease diagnoses are difficult to make and confounded by a number of genetic and lifestyle factors, there are conflicting reports in reference to the nosological status, prevalence, diagnostic criteria and etiopathogenesis of this neurodegenerative disorder (Sachdeva et al., 2016). Studies correlating the amount of alcohol consumed and the duration of consumption to the occurrence of alcohol related neurodegenerative disease (ARD) are not yet available (Sachdeva et al., 2016), and comprise an essential future step in understanding the impact of alcohol on dementia risk.

There are a number of neurotransmitter systems involved in the effects of alcohol in the brain. Alcohol in high doses can have sedative and anxiolytic effects mediated through GABAergic activity and inhibition of excitatory neurotransmitters, such as, glutamate (Wiegmann et al., 2020). In alcohol withdrawal, the rebound effects in the GABAergic and glutamatergic systems are compounded by monoamine dysfunction (Wiegmann et al., 2020). A proposed mechanism behind alcohol-induced neuronal damage is the excitotoxicity that accompanies excessive neurotransmitter release (Tiwari et al., 2009). The excessive activation of NMDA receptors and absence of GABAergic inhibition during withdrawal from alcohol may heighten the localized vulnerability of neurons consistent with that of degenerative neuropathology (Hynd et al., 2004). l-glutamate (l-Glu), an excitatory amino acid, may underlie the pathogenic mechanisms of alcohol in chronic neurodegenerative disorders that produce dementia (Hynd et al., 2004).

Individuals who have been diagnosed with alcoholism are likely to have frontal lobes that are particularly susceptible to alcohol-induced damage (Ridley et al., 2013). They evidence noticeably decreased neuron density, modified glucose metabolism and perfusion, as well as total volume shrinkage (Ridley et al., 2013). Prominent white matter loss and neuronal loss in the superior frontal association cortex, cerebellum and hypothalamus have also been identified via neuroimaging (Ridley et al., 2013). Through abstinence, partial recovery of the white matter tracts can occur (Ridley et al., 2013). In the central nervous system, high concentrations of alcohol activates oxidases and elevates homocysteine (Wiegmann et al., 2020). These are the first steps in alcohol-related brain damage. Chronic alcohol consumption drives neuronal damage through increased production of reactive oxygen species and oxidative-nitrosative stress (Tiwari et al., 2009), as previously described. This can trigger an inflammatory cascade that eventually results in neuronal apoptosis and finally the symptoms of dementia (Tiwari et al., 2009). Several explanations for the mechanisms behind alcohol-induced selective neuronal damage have been proposed, although the mechanisms themselves are not well understood (Tiwari et al., 2009). An example would be edema caused by modifications in cellular control of ion transport (Tiwari et al., 2009). Chronic exposure to alcohol is also followed by the translocation of protein kinase C (PKC) and the activation of NFkβ and PKC, which precede DNA fragmentation (Tiwari et al., 2009). DNA fragmentation then drives neuronal death through apoptosis and other mechanisms, leading to the neurodegeneration that underlies behavioral decline as well as dementia (Tiwari et al., 2009). Though there are numerous studies that support the occurrence of alcoholic dementia and neurodegeneration, more work is needed to extend these findings beyond the role of alcohol metabolism in oxidative stress.

Long-term misuse of alcohol frequently results in vitamin B1 (thiamine) deficiencies that can also lead to brain damage. Alcohol, combined with the malnutrition that occurs when too many calories are obtained from alcohol rather than a balanced diet, can interfere with the absorption of vitamins including thiamine. Low-level deficiencies in thiamine are often unrecognized in alcoholic patients and they can end up being nutritionally depleted for extended periods of time before symptoms of vitamin deficiency manifest clinically (Peter et al., 2003). The clinical manifestation of these symptoms, Korsakoff syndrome (KS), is often associated with Wernicke’s encephalopathy (WE) which is a neurodegenerative disorder caused by the extended absence of thiamine in the diet 'Wernicke-Korsakoff Syndrome Information Page', 2019). The individual who gave the first comprehensive description of a mental disturbance in alcoholics and non-alcoholics characterized by retentive memory and frequent association with polyneuropathy was S.S. Korsakoff, a Russian physiatrist (Victor, 1994). The signature symptom of KS is anterograde amnesia, or the inability to create new memories after the event that caused the degeneration (Oscar-Berman, 2012). There are also other symptoms like confabulation (Oscar-Berman, 2012), impaired executive function (Oscar-Berman, 2012; Ridley et al., 2013), and visuoperceptual difficulties (Ridley et al., 2013). The pathology of KS has been described as lesions in the diencephalon-hippocampal circuit, inside the midline thalamus and anterior thalamic nuclei (Wiegmann et al., 2020). Greater loss of neurons in the nucleus basalis has been seen in individuals who are diagnosed with KS and ARD as opposed to those with uncomplicated alcoholism, though this needs replication for further analysis (Ridley et al., 2013). Most studies report that cognitive function remains broadly intact because memories formed before the onset of prolonged heavy drinking remain preserved in cortical regions, whereas working memory and consolidation of new memories are both impacted by this form of neurodegeneration (Oscar-Berman, 2012).

There is still some controversy in the literature about the definition of alcohol-related dementia and whether or not it represents an entity distinct from KS (Wiegmann et al., 2020). There is no consensus, and while the neuropsychological findings from each diagnosis may encompass unique cortical and subcortical patterns (Wiegmann et al., 2020), the findings are not robust enough for a clear delineation between them.

Frontotemporal dementia (FTD) can be characterized by a wide variety of abnormal responses to reward stimuli, suggesting altered functioning of brain circuitry mediating reward processing. Examples include food, intoxicants, and sex (Perry et al., 2014b). Because of the prominent behavioral features of FTD, it can mimic other types of psychiatric disorders (Bang et al., 2015). FTD is considered a broad categorization encompassing multiple neurodegenerative disease with heterogeneous clinical presentations (Tsai and Boxer, 2014). Partially overlapping regions of the reward circuit, particularly in the right hemisphere, influence each of the reward-related behaviors (Perry et al., 2014a). These regions include the portions of the basal ganglia (thalamus, globus pallidus, putamen) and insula (Perry et al., 2014a). The progressive decline of behavior, cognition, and personality results from prominent frontal lobar atrophy (Graham et al., 2005) that extends to the temporal lobe as the disease progresses. Thus, frontal symptoms—such as, behavioral disinhibition—tend to precede cognitive symptoms, whereas the opposite pattern is seen in Alzheimer’s disease (Kelley and Petersen, 2007). While FTD is less common than Alzheimer’s disease, it makes up 50% of dementia cases presenting before age 60 (Götz and Ittner, 2008).

There are three defined clinical syndromes that encompass FTD: semantic variant primary progressive aphasia (svPPA), behavioral variant frontotemporal dementia (bvFTD), and nonfluent variant primary progressive aphasia (nfvPPA) (Tsai and Boxer, 2014). Aphasia is a disorder that impairs the expression and understanding of language due to damage to the regions of the brain that oversee language 'Aphasia', 2015), typically the left side. In this case, FTD as a whole will be discussed. Alcohol's influence and the part it plays in FTD is not well studied and there is not much evidence supporting the idea that heavy alcohol consumption or alcoholism leads to FTD.

Some core clinical diagnostic features of individuals with FTD are early loss of personal awareness, hyperorality which can include excessive smoking and alcohol consumption, and early signs of disinhibition 'Clinical and neuropathological criteria for frontotemporal dementia. The Lund and Manchester Groups' 1994). Synapse loss in the superior laminae of the frontal cortical area is more likely attributed to alcohol and may be mediated through vitamin B deficiency (Brun and Andersson, 2001). These changes are similar to those observed in FTD. This seems to be a possible main cause of alcoholic dementia and the alcoholic frontal symptomatology (Brun and Andersson, 2001). Although one paper in 2003 failed to find an association of alcohol use in FTD risk, less than 10% of the very small sample size (8 FTD patients and 7 controls) has a history of heavy alcohol intake (Rosso et al., 2003). Just as the symptoms of FTD can be heterogeneous, so can the pathologies associated with the various subtypes. Tau-positive or TAR DNA-binding protein 43 (TDP-43)-positive inclusion bodies are involved in the majority of pathologies accompanying FTD clinical syndromes (Rabinovici and Miller, 2010). There is a clinical and pathological overlap between FTD, corticobasal degeneration, progressive supranuclear palsy, and amyotrophic lateral sclerosis (Rabinovici and Miller, 2010). A significant subset of patients with FTD develop late-onset parkinsonism and display asymmetrical atrophy of the temporal and frontal cortex (Götz and Ittner, 2008). While the symptoms of FTD can be heterogeneous, the pathological and clinical heterogeneity poses a notable diagnostic challenge (Rabinovici and Miller, 2010).

### Ataxias

Although alcohol can contribute to other types of ataxia, its most potent and most common effects are on cerebellar ataxia. Alcohol produces both acute and long-term effects on cerebellar function, and can be both the primary or a secondary cause of cerebellar neuron degeneration (Shanmugarajah et al., 2016), especially in the white matter (Zhao et al., 2020). This is most easily imagined as the so-called ‘alcoholic gait’ in which an intoxicated person displays an unsteady, often staggering, stance as they walk, although unsteady upper limb movements can also occur with extreme intoxication. These deficits, as well as decreases in cerebellar volume, can persist well into alcohol abstinence (Sullivan and Pfefferbaum, 2005) and are especially prominent following prenatal alcohol exposure (West, 1993; (Sullivan et al., 2020; Taggart et al., 2017). Despite this evidence, and the common clinical presentation of ataxia with a history of risky alcohol exposure, the mechanisms underlying neurodegeneration are still being elucidated. One recognized cause, which is outside of the scope of this review, is the metabolic disturbance caused by alcoholic liver disease, which can damage the brain through the brain-liver axis (Chen et al., 2012; de la Monte et al., 2009). Indeed, the peripheral damage caused by alcohol exposure is associated with an increase in inflammation in the brain (Poole et al., 2017), and the neurodegeneration caused by alcoholic liver disease is driven by astrocytosis in the diencephalon (thalamus and hypothalamus) and the cerebellum (Kril and Butterworth, 1997). However, this review will focus on direct neuronal damage due to alcohol exposure. The most well-studied mechanism of alcohol-induced neurodegeneration is thiamine deficiency. Thiamine, or vitamin B1, is an essential coenzyme in human metabolism that can only be acquired through diet. Many chronic alcoholics replace dietary calories with alcohol calories, and thiamine intake is reduced along with dietary intake. Thiamine deficiency then drives oxidative stress and endoplasmic reticulum stress, which both disrupt homeostatic autophagy processes (Liu D. et al., 2017). Alcohol also increases oxidative stress and endoplasmic reticulum stress (Haorah et al., 2005; Wang et al., 2018), and recent work has demonstrated that alcohol and thiamine deficiency have separate but additive effects on these processes in the brain (Matsui et al., 2012; He et al., 2007). Alcohol also reduces thiamine absorption when thiamine levels are low (Hoyumpa, 1980), complicating interpretation in clinical studies of chronic alcoholism and neurodegeneration in most brain regions. However, the cerebellum is especially sensitive to alcohol-induced damage, such that even moderate alcohol intake in the absence of thiamine deficiency can cause degeneration in the vermis of the cerebellum, the region of the spinocerebellum that coordinates axial movements (Karhunen et al., 1994). This degeneration underlies truncal ataxia, or the instability of the axial muscles.

Although alcohol-induced neuronal stress is likely to reduce the autophagic clearance of toxic intracellular proteins throughout the brain, some evidence suggests that this mechanism of neurodegeneration is more prominent in the cortex and hippocampus (Davis-Anderson et al., 2018; Liu et al., 2019; Topper et al., 2015). Alcohol also damages the cerebellum by driving microglia to a hyperactive, pro-inflammatory state. In a rat model of prenatal alcohol exposure, cerebellar microglia increased in density and showed a higher number in the amoeboid shape that indicates a pro-inflammatory, destructive state (Gursky et al., 2020; Topper et al., 2015). This suggests that inflammation-associated neuron loss is higher in the alcohol-exposed cerebellum. In addition, alcohol withdrawal, and the consequent glutamatergic excitotoxicity and upregulation of stress-related p38 kinase, has also been shown to damage cerebellar Purkinje cells (Virmani et al., 1985; Netzeband et al., 2002). Glutamate excitotoxicity is associated with increased Ca+2 influx into neurons; this calcium is normally bound by parvalbumin in order to maintain homeostasis, and alcohol withdrawal is associated with a loss of parvalbumin-expressing cerebellar Purkinje cells in the cerebellum with less effect in the hippocampus and cortex (Rewal et al., 2005). Alcohol withdrawal also increases the permeability of mitochondria to Ca+2 influx and increases the opening of the nonspecific permeability transition pore, which causes efflux of mitochondrial signals that induce neuron death (Rewal et al., 2005). At the same time, alcohol reduces the expression of inhibitory GABAA receptors, glutamic acid decarboxylase, which catalyzes the metabolism of glutamate to GABA (Proudfoot and Wilkins, 2017), and the protein that promotes GABA uptake into synaptic vesicles (Gruol et al., 2020), further pushing the signaling balance toward a hyperexcitatory state. Interestingly, the actions of alcohol that evoke glutamate excitotoxicity differentiate alcohol-induced cerebellar ataxia from many other forms, which have been shown to depend on glutamate excitotoxicity through a deficiency in the expression of glutamic acid decarboxylase, which catalyzes the metabolism of glutamate to GABA (Proudfoot and Wilkins, 2017).

Recent studies have elucidated proteomic changes induced by alcohol that might contribute to these mechanisms of cerebellar ataxia as well. Chronic alcohol exposure downregulates the expression of microtubule-associated protein-2 (MAP-2), a key regulator of synaptic plasticity and neuron structural integrity (Abulaiti et al., 2016). Interestingly, MAP-2 is heavily expressed throughout the cerebellum across aging (Tucker et al., 1988), whereas its expression decreases with age in the hippocampus, and this decrease is associated with cognitive loss (Di Stefano et al., 2001). Thus, loss of MAP-2 in the cerebellum is likely to reduce the plasticity required for normal motor learning and coordination. Alcohol exposure also increases the expression of voltage-dependent anion channel type 1 (VDAC1) a Ca+2 channel and essential component of the permeability transition pore that mediates mitochondrial damage-induced neuron death in alcohol withdrawal (Abulaiti et al., 2016). Fetal alcohol exposure is also associated with cerebellum-specific downregulation of proteins associated with axon growth and regulation of ion—including Ca+2—flux, as well as with a brain-wide downregulation of the autophagy mediator Rab-21 (Davis-Anderson et al., 2018).

Frontal ataxia (Bruns apraxia) is less common than cerebellar ataxia and even the categorization of this ataxia is controversial. Indeed, the etiology—damage to the frontal lobe, including the basal ganglia and adjacent white matter tracts—gives rise to a Parkinsonism difficulty in initiating the motor movements necessary for basic functions, such as, speaking and walking. Thus, it may be better categorized as an apraxia, or inability to perform a skilled motor task despite the comprehension and desire to perform the given task. In almost all cases resulting in frontal ataxia/apraxia, damage to the superior longitudinal fasciculus and frontostriatal network is the causative factor (Pramstaller and Marsden, 1996). These white matter tracts connect the cortical executive function areas with cortical association areas and motor output areas.

While the white matter damage associated with frontal ataxia may be caused by any number of lesions (e.g. infarction, tumor, hemorrhage), or even genetic disorders, such as, Down Syndrome (Powell et al., 2014), they are frequently exacerbated by alcohol exposure. The structural integrity of the superior longitudinal fasciculus is diminished in patients with a history of heavy alcohol consumption (Monnig et al., 2014), adolescents who are currently abusing alcohol (Bava et al., 2013), and neonates and older children who were prenatally exposed to alcohol (Donald et al., 2015). Together, these data suggest that any history of exposure to high alcohol level will increase the susceptibility of the superior longitudinal fasciculus to further insults that result in frontal ataxia. Similarly the frontostriatal network demonstrates a deficit in functional connectivity in patients with a history of adult (Broadwater et al., 2018) or adolescent alcohol abuse (Galandra et al., 2019), while prenatal alcohol produces alterations in connectivity, enhancing fronto-putamen connections to the detriment of fronto-caudate connections (Roussotte et al., 2012). In addition, work in the field of alcohol addiction has demonstrated that alcohol shifts energetics in the brain from glucose metabolism to acetate metabolism (Volkow et al., 2015), which may produce more direct effects on neuron integrity and survival.

### Niemann-Pick Disease

Niemann-Pick disease consists of two subtypes with a common mechanism—Types A and B—and the completely unique Type C. In Types A and B, a mutation in the gene encoding the enzyme acid sphingomyelinase leads to deficits in sphingomyelin metabolism and lipid storage, with subsequent foam cell infiltration. Type A is the pediatric type, which generally results in mortality by three years of age. While fetal alcohol exposure may contribute to disease etiology, its universal juvenile lethality (McGovern et al., 2006) puts it outside the scope of this review. Type B is the adult type; although patients are still most likely to be diagnosed in early childhood, a mutation with a milder phenotype allows a closer-to-normal life expectancy. In approximately 30% of cases of Neimann-Pick Type B, individuals with a Type B variant—alternately referenced as Type A/B or Type E—have a specific mutation in acid sphingomyelinase (Q292K) that is associated with severe neurodegeneration and neurobehavioral phenotypes (Pavlů-Pereira et al., 2005; Wasserstein et al., 2006).

Most of the research that has examined the interactions between sphingomyelinase and alcohol has focused on the mediation of alcohol reward and positive affective drive by sphingolipids (Müller et al., 2017; Kalinichenko et al., 2019a). But there is also evidence that alcohol exposure and withdrawal have potent effects on the activity of acid sphingomyelinase itself. Acid sphingomyelinase is upregulated in response to acute alcohol exposure and in alcohol-dependent patients, although this upregulation gradually returns to normal levels during alcohol withdrawal (Reichel et al., 2010; Reichel et al., 2011). However, both the acute alcohol-induced increase in acid sphingomyelinase, and the withdrawal-induced decrease in the same, are greater in males (Mühle et al., 2014), suggesting possible sex-dependent effects in related diseases. The paucity of work in this area, combined with differences in age and sex distribution between studies, makes it difficult to draw specific conclusions about the effects of alcohol abuse of long-term acid sphingomyelinase activity. Nonetheless, the direct effects of alcohol exposure on acid sphingomyelinase, and the key neurological signs in the Neimann-Pick Type B variant, suggest that patients with a mutation in the gene encoding acid sphingomyelinase will be at greater risk if they become alcohol-dependent. Furthermore, the highly variable natural course of the disease—in which some individuals are not diagnosed until late adulthood—increases the likelihood that alcohol exposure will play a factor in the etiology of Niemann-Pick Type B variant. However, both basic science and clinical research is needed to investigate this possibility.

In Niemann-Pick Type C, the causative mutation is in one of two genes that encode lysosomal transport proteins, NPC1 and NCPC2, and both mutations lead to unesterified cholesterol accumulation in lysosomes due to a deficit in cholesterol transport (Vázquez et al., 2012; Vanier and Marie, 2010; Chen et al., 2001). Like Type A, Type C is most frequently characterized as a pediatric disorder, but there are 2–3 times more cases of adolescent and adult onset (Yanjanin et al., 2010; Wassif et al., 2016). However, Type C is primarily a neurodegenerative disorder, and its diagnosis is often confounded by neurological signs common to a variety of diseases (Balázs et al., 2019; Rego et al., 2019; Johnen et al., 2018). The signs include vertical gaze palsy, cerebellar ataxia, and dystonia, as well as mild cognitive deficits in visual memory, verbal fluency, and executive function. And Niemann-Pick Type C is often misdiagnosed as early onset Alzheimer’s disease—both diseases present with amyloidosis and tau tangles, as well as with deficits in synaptic plasticity.

The mechanisms by which neurocognitive function is impaired in Niemann-Pick Type C have recently begun to be elucidated, and these mechanisms are likely to be impacted by a history of alcohol abuse. First, changes in both autophagy and oxidative stress both play a role in the cellular damage seen in the disease (Vázquez et al., 2012; Sarkar et al., 2013); the effects of alcohol on these pathways has already been discussed in detail in preceding sections. Second, there are noted deficits in cholinergic signaling in Niemann-Pick Type C patients (Benussi et al., 2019); alcohol also has a potent effect on cholinergic activity. Intermittent, or binge, exposure to alcohol in adolescence reduces the number of neurons that express choline acetyltransferase, the enzyme that is responsible for the production of acetylcholine (Swartzwelder et al., 2015). In the adult brain, cholinergic varicosities—the axonal structures that release neurotransmitters—are similarly reduced by chronic alcohol (Pereira et al., 2014). Even prenatal alcohol alters cholinergic signaling, although in the case of developmental alcohol, the shift is in the density of specific receptor subtypes (Monk et al., 2012). Thus, chronic exposure to alcohol at any age is likely to exacerbate or even precipitate the onset of Niemann-Pick Type C symptoms. The third mechanism that is likely impacted by alcohol in Niemann-Pick Type C is through epigenetic modification. There are decreases in DNA methyltransferase expression and in global DNA methylation in a mouse model of Niemann-pick Type C (with the NPC1 mutation) (Kennedy et al., 2016). And histone deacetylase (HDAC) inhibitors have proven efficacy in reducing cholesterol accumulation in patients with Niemann-Pick Type C (Helquist et al., 2013). Thus, changes in methylation and acetylation are likely to be major factors in the progression of the disease. As discussed in prior sections, alcohol produces potent effects on the epigenome as well. Although acute alcohol actually inhibits HDACs itself, alcohol withdrawal produces a robust increase in HDAC activity (Pandey et al., 2008), which could precipitate the symptoms of Niemann-pick Type C. And alcohol produces a host of effects on DA methylation, through generation of acetaldehyde, DNA damage, and through deficiencies in the vitamin B and folate that are necessary for the production of S-adenosyl-methionine, the methyl group donor for DNA methylation (Ponomarev, 2013). There are likely a host of other epigenetic mechanisms that may be common to Niemann-Pick Type C and alcohol abuse, but these connections remain to be elucidated.

## Conclusions and Future Directions

In summary, despite a wealth of circumstantial evidence for an important role of alcohol exposure in the etiology of rare neurodegenerative disorders, a great deal of research remains to be done to test these connections directly. For example, alcohol may not have similar effects across different brain regions, considering variability in neuron type, glial cell density, and other factors. Furthermore, there is a heavy reliance on data from studies of alcohol abuse in risk of the most common neurodegenerative diseases—especially Alzheimer’s disease and Parkinson’s disease—while the reality is likely to reflect unique effects of alcohol in each of the neurodegenerative diseases because each has its own etiological and pathophysiological profile. Furthermore, because alcohol is such a dirty drug—affecting the central nervous system at the genetic and epigenetic, molecular, and cellular levels—it is essential to identify the mechanisms that underlie the long-term, most neurodegenerative effects of alcohol. Nonetheless, the data that have been gathered thus far suggest that reducing alcohol abuse, or pharmacologically moderating the physiological, neuronal effects of alcohol may be a worthwhile therapeutic goal in individuals at increased risk of developing these diseases.
